# EANM practice guideline/SNMMI procedure standard for dopaminergic imaging in Parkinsonian syndromes 1.0

**DOI:** 10.1007/s00259-020-04817-8

**Published:** 2020-05-09

**Authors:** Silvia Morbelli, Giuseppe Esposito, Javier Arbizu, Henryk Barthel, Ronald Boellaard, Nico I. Bohnen, David J Brooks, Jacques Darcourt, John C. Dickson, David Douglas, Alexander Drzezga, Jacob Dubroff, Ozgul Ekmekcioglu, Valentina Garibotto, Peter Herscovitch, Phillip Kuo, Adriaan Lammertsma, Sabina Pappata, Iván Peñuelas, John Seibyl, Franck Semah, Livia Tossici-Bolt, Elsmarieke Van de Giessen, Koen Van Laere, Andrea Varrone, Michele Wanner, George Zubal, Ian Law

**Affiliations:** 1 IRCCS Ospedale Policlinico San Martino, Genoa, Italy; 2 Nuclear Medicine Unit, IRCCS Ospedale Policlinico San Martino, Department of Health Sciences, University of Genoa, Genoa, Italy; 3 Nuclear Medicine, Department of Radiology, Medstar Georgetown University Hospital, 3800 Reservoir Road NW CCC Bldg, Washington, DC 20007 USA; 4 Department of Nuclear Medicine, University of Navarra, Clinica Universidad de Navarra, IdiSNA, Pamplona, Spain; 5 Department of Nuclear Medicine, Leipzig University, Leipzig, Germany; 6 Department of Radiology and Nuclear Medicine, Amsterdam UMC, Vrije Universiteit Amsterdam, NeuroScience, De Boelelaan 1117, Amsterdam, The Netherlands; 7 Departments Radiology & Neurology, University of Michigan & Ann Arbor Veterans Medical Center, Ann Arbor, MI USA; 8 Department of Nuclear Medicine, Aarhus University, Aarhus, Denmark; 9 Institute of Neuroscience, University of Newcastle upon Tyne, Newcastle upon Tyne, UK; 10 Nuclear Medicine, Department of imaging, Centre Antoine Lacassagne, Université Cote d’Azur, Nice, France; 11 Institute of Nuclear Medicine, University College London Hospitals NHS Foundation Trust, London, UK; 12 Department of Nuclear Medicine and Neuroradiology, Stanford University, Stanford, CA USA; 13 Department of Nuclear Medicine, Faculty of Medicine and University Hospital Cologne, University of Cologne, Cologne, Germany; 14 German Center for Neurodegenerative Diseases (DZNE), Bonn-Cologne, Germany; 15 Molecular Organization of the Brain, Research Center Jülich, Institute of Neuroscience and Medicine (INM-2), Jülich, Germany; 16 Division of Nuclear Medicine and Clinical Molecular Imaging in the Department of Radiology, Perelman School of Medicine at the University of Pennsylvania, Philadelphia, PA USA; 17 Department of Nuclear Medicine, Sisli Etfal Education and Research Hospital, Istanbul, Turkey; 18 NIMTLab, Faculty of Medicine, Geneva University, Geneva, Switzerland; 19 Division of Nuclear Medicine and Molecular Imaging, Geneva University Hospitals, Geneva, Switzerland; 20 Positron Emission Tomography Department, NIH/CC, Bethesda, MD USA; 21 Departments of Medical Imaging, Medicine and Biomedical Engineering, University of Arizona, Tucson, AZ USA; 22 National Council of Research, Institute of Biostructure and Bioimaging, Naples, Italy; 23 Institute for Neurodegenerative Disorders, New Haven, USA; 24 Nuclear Medicine Department, University Hospital, Lille, France; 25 Medical Physics, University Hospital Southampton NHS Foundation Trust, Southampton, UK; 26 Radiology and Nuclear Medicine, Amsterdam UMC, University of Amsterdam, Meibergdreef 9, Amsterdam, The Netherlands; 27 Nuclear Medicine and PET-MR Unit, Department of Imaging and Pathology, University Hospital and KU Leuven, Leuven, Belgium; 28 Centre for Psychiatry Research, Department of Clinical Neuroscience, Karolinska Institutet & Stockholm Health Care Services, Region Stockholm, Stockholm, Sweden; 29 University of Washington, Seattle, WA USA; 30 Nuclear Medicine and CT, NIH/NIBIB, Bethesda, USA; 31 Department of Clinical Physiology, Nuclear Medicine and PET, Rigshospitalet, University of Copenhagen, Copenhagen, Denmark

**Keywords:** Brain, DAT, DOPA, PET, SPECT, Parkinson, Parkinsonian syndromes

## Abstract

**Purpose:**

This joint practice guideline or procedure standard was developed collaboratively by the European Association of Nuclear Medicine (EANM) and the Society of Nuclear Medicine and Molecular Imaging (SNMMI). The goal of this guideline is to assist nuclear medicine practitioners in recommending, performing, interpreting, and reporting the results of dopaminergic imaging in parkinsonian syndromes.

**Methods:**

Currently nuclear medicine investigations can assess both presynaptic and postsynaptic function of dopaminergic synapses. To date both EANM and SNMMI have published procedural guidelines for dopamine transporter imaging with single photon emission computed tomography (SPECT) (in 2009 and 2011, respectively). An EANM guideline for D2 SPECT imaging is also available (2009). Since the publication of these previous guidelines, new lines of evidence have been made available on semiquantification, harmonization, comparison with normal datasets, and longitudinal analyses of dopamine transporter imaging with SPECT. Similarly, details on acquisition protocols and simplified quantification methods are now available for dopamine transporter imaging with PET, including recently developed fluorinated tracers. Finally, [^18^F]fluorodopa PET is now used in some centers for the differential diagnosis of parkinsonism, although procedural guidelines aiming to define standard procedures for [^18^F]fluorodopa imaging in this setting are still lacking.

**Conclusion:**

All these emerging issues are addressed in the present procedural guidelines for dopaminergic imaging in parkinsonian syndromes.

## Preamble

The Society of Nuclear Medicine and Molecular Imaging (SNMMI) is an international scientific and professional organization founded in 1954 to promote the science, technology, and practical application of nuclear medicine. Its 15,000 members are physicians, technologists, and scientists specializing in the research and practice of nuclear medicine. In addition to publishing journals, newsletters, and books, the SNMMI also sponsors international meetings and workshops designed to increase the competencies of nuclear medicine practitioners and to promote new advances in the science of nuclear medicine. The European Association of Nuclear Medicine (EANM) is a professional nonprofit medical association that facilitates communication worldwide between individuals pursuing clinical and research excellence in nuclear medicine. The EANM was founded in 1985.

The SNMMI/EANM periodically define new standards/guidelines for nuclear medicine practice to help advance the science of nuclear medicine and to improve the quality of service to patients. Existing standards/guidelines will be reviewed for modifications or renewal, as appropriate, on their fifth anniversary or sooner, if indicated. As of February 2014, the SNMMI guidelines are referred to as procedure standards. Any previous practice guideline or procedure guideline that describes how to perform a procedure is now considered an SNMMI procedure standard.

Each standard/guideline, representing a policy statement by the SNMMI/EANM, has undergone a thorough consensus process in which it has been subjected to extensive review. The SNMMI/EANM recognizes that the safe and effective use of diagnostic nuclear medicine imaging requires specific training, skills, and techniques, as described in each document.

The EANM and SNMMI have written and approved these standards/guidelines to promote the use of nuclear medicine procedures with high quality. These standards/guidelines are intended to assist practitioners in providing appropriate nuclear medicine care for patients. They are not inflexible rules or requirements of practice and are not intended, nor should they be used, to establish a legal standard of care. For these reasons and those set forth below, the SNMMI/EANM cautions against the use of these standards/guidelines in litigation in which the clinical decisions of a practitioner are called into question.

The ultimate judgment regarding the propriety of any specific procedure or course of action must be made by medical professionals considering the unique circumstances of each case. Thus, there is no implication that an approach differing from the standards/guidelines, standing alone, is below the standard of care. To the contrary, a conscientious practitioner may responsibly adopt a course of action different from that set forth in the standards/guidelines when, in the reasonable judgment of the practitioner, such course of action is indicated by the condition of the patient, limitations of available resources, or advances in knowledge or technology subsequent to publication of the standards/guidelines.

The practice of medicine involves not only the science but also the art of dealing with the prevention, diagnosis, alleviation, and treatment of disease. The variety and complexity of human conditions make it impossible to always reach the most appropriate diagnosis or to predict with certainty a particular response to treatment. Therefore, it should be recognized that adherence to these standards/guidelines will not ensure an accurate diagnosis or a successful outcome. All that should be expected is that the practitioner will follow a reasonable course of action based on current knowledge, available resources, and the needs of the patient to deliver effective and safe medical care. The sole purpose of these standards/guidelines is to assist practitioners in achieving this objective.

The present guideline/standard was developed collaboratively by the EANM and SNMMI. It summarizes the views of the Neuroimaging Committee of the EANM and the Brain Imaging Council of the SNMMI and reflects recommendations for which the EANM and SNMMI cannot be held responsible. The recommendations should be taken into context of good practice of nuclear medicine and do not substitute for national and international legal or regulatory provisions.

## Introduction

Parkinsonian syndromes are a group of diseases characterized by signs of parkinsonism, such as bradykinesia, rigidity, tremor, and postural instability. Idiopathic Parkinson’s disease (IPD) is the most common cause of parkinsonism, but several other etiologies result to the presence of parkinsonism. Indeed, parkinsonism can be present in all alpha synucleinopathies, which include Lewy body diseases (LBDs), a subset of disorders associated with the accumulation of Lewy bodies (LB) and neurites, i.e., intracytoplasmic inclusions composed of aggregated alpha-synuclein and other proteins such as ubiquitin [1]. The most clinically relevant subtypes of LBDs are IPD and dementia with Lewy bodies (DLB) [1]. Alpha synucleinopathies also include multiple system atrophy (MSA), an atypical parkinsonism characterized by the presence of glial and neural silver staining aggregates of alpha-synuclein [2]. Finally, the tauopathies corticobasal degeneration (CBD) and progressive supranuclear palsy (PSP) are movement disorders belonging to the spectrum of frontotemporal degeneration (and they are also defined as atypical parkinsonisms) [3, 4]. The differential diagnosis of the neurodegenerative parkinsonian syndromes includes clinical entities such as essential tremor (ET), drug-induced parkinsonism, vascular parkinsonism, and psychogenic parkinsonism. ET is characterized by the presence of tremor during voluntary movement rather than at rest. Resting tremor and cogwheel rigidity or other isolated parkinsonian characteristics can be present in a subgroup of patients with ET, thus making the clinical diagnosis more challenging [5].

In many patients, the clinical differential diagnosis of parkinsonism is relatively straightforward [1]. However in numerous conditions, an improvement in diagnostic accuracy is possible using dopaminergic imaging [6]. This imaging technology may be particularly helpful in patients with incomplete or atypical syndromes, unsatisfying response to therapy, and overlapping symptoms or in patients with early/mildly symptomatic stages of disease.

Currently, nuclear medicine investigations can assess both presynaptic and postsynaptic function of dopaminergic synapses [6–8]. Presynaptic dopaminergic imaging helps clarify the differential diagnosis between neurodegenerative parkinsonian syndromes and non-dopamine deficiency etiologies of parkinsonism [6–8].

Presynaptic dopaminergic function can be summarized as follows. Dopamine is produced via two amino acids. First, L-tyrosine is hydroxylated to form L-dopa, which subsequently is decarboxylated to dopamine by aromatic L-amino-acid decarboxylase (AADC; also known as dopa decarboxylase). Next, dopamine is transported to intracellular vesicles by vesicular monoamine transporter 2 (VMAT2). As a result of neuronal depolarization, these vesicles are emptied into the synaptic cleft where the synaptic dopamine then interacts with postsynaptic dopamine receptors. After dopamine has been emptied into the synaptic cleft, it can be subject to reuptake into the presynaptic neuron by dopamine transporter (a.k.a. dopamine active transporter—DAT).

Postsynaptic dopamine receptors can be divided into D_1_-like receptors (D_1_, D_5_) and D_2_-like receptors (D_2_, D_3_, and D_4_) [9]. Over 90% of D_2_ receptors are located postsynaptically and so imaging of D_2_ receptors is frequently referred to as imaging of postsynaptic D_2_ receptors [10]. To date, three procedure guidelines/procedure standards have been published by EANM and SNMMI, respectively. Both EANM and SNMMI have published guidelines/standards for dopamine transporter imaging with SPECT (in 2009 and 2011, respectively) An EANM Guideline for D_2_ SPECT imaging is also available (2009) [9, 11–13]. Since the publication of these previous documents, new lines of evidence have been made available on semiquantification, harmonization, comparison with normal datasets, and longitudinal analyses of DAT SPECT. Similarly, details on acquisition protocols and simplified quantification methods are now available for dopamine transporter imaging with PET, including recently developed fluorinated tracers. Finally, especially in some nuclear medicine centers equipped with PET and without cyclotron, [^18^F]fluorodopa PET is performed for in patients with parkinsonism, although procedural GLs aiming to define standard procedures for [18F]fluorodopa imaging in this setting are still lacking.

Goals.

The aim of this guideline is to assist nuclear medicine physicians in recommending, performing, interpreting, and reporting the results of dopaminergic imaging in parkinsonian patients.

## Definitions

1. SPECT—single photon emission computed tomography (also known as SPET). It allows imaging of the three dimensional distribution of radiopharmaceuticals labeled with gamma-ray emitting radionuclide such as ^123^I and ^99^mTc.
2. SPECT/CT—SPECT imaging combined with CT (computed tomography) in a hybrid scanner.
3. Positron emission tomography. This imaging modality is specific for imaging positron-emitting radionuclides such as ^11^C and ^18^F through coincidence detection of annihilation photon pairs.
4. PET/CT—PET imaging combined with CT (computed tomography) in a hybrid scanner. PET may also be combined with magnetic resonance imaging (MRI); however, the use of these scanners in patients with movement disorders has not yet been adequately addressed in the literature.
5. Presynaptic dopaminergic imaging refers to SPECT and PET imaging studies evaluating the integrity of presynaptic nigrostriatal dopaminergic synapses.
6. Postsynaptic dopaminergic imaging refers to SPECT and PET imaging studies that evaluate the integrity of dopaminergic neurons at the postsynaptic level (frequently referred to as imaging of the postsynaptic D2 receptors).

## Clinical indications

Presynaptic dopaminergic imaging is indicated for detecting loss of nigrostriatal dopaminergic neuron terminals of patients with parkinsonian syndromes, especially:

- To support the differential diagnosis between essential tremor and neurodegenerative parkinsonian syndromes. Note that presynaptic dopaminergic imaging is unable to distinguish IPD and DLB from PSP, CBD, or putaminal variant of MSA [14–17].
- To help distinguish between DLB and other dementias (in particular, Alzheimer’s disease, AD) [18–20].
- To support the differential diagnosis between parkinsonism due to presynaptic degenerative dopamine deficiency and other forms of parkinsonism, e.g., between IPD and drug-induced, psychogenic, or vascular parkinsonism [21–23].
- To detect early presynaptic parkinsonian syndromes [24, 25].

Postsynaptic dopaminergic imaging can help separate typical from atypical parkinsonian syndromes. The main indication is the differentiation of IPD from other neurodegenerative parkinsonian syndromes where loss of D2 receptors occurs (e.g., MSA, PSP) [26]. However, the clinical use of SPECT or PET tracers for postsynaptic dopaminergic imaging is currently limited by several factors and has been in many centers replaced by other molecular imaging targets [27, 28] (see section IX).

Less common indications for postsynaptic dopaminergic imaging in parkinsonian syndromes:

1. Assessment of the extent of D2 receptor blockade during treatment with dopamine D2 antagonists (neuroleptics).
2. Wilson’s disease. Loss of striatal D2 receptor function is related to the severity of neurological symptoms in Wilson’s disease and may show the degree of neuronal damage due to cytotoxic copper deposition in the striatum.

## Qualification and responsibilities of the personnel

### Physician

SPECT and PET examinations should be performed by or under supervision of a physician specialized in nuclear medicine and certified by accrediting boards. In Europe, the certified nuclear medicine physician who authorizes the study and signs the report is responsible for the procedure, according to national laws and rules. For the USA, see the SNMMI Guideline for General Imaging (Society of Nuclear Medicine., 2010, http://interactive.snm.org/docs/General_ Imaging_Version_6.0.pdf).

### Technologist

SPECT and PET examinations should be executed by qualified registered/certified Nuclear Medicine Technologists. Please refer to: Performance Responsibility and Guidelines for Nuclear Medicine Technologists 3.1 and http://www.eanm.org/content-eanm/uploads/2016/11/EANM_2017_TC_Benchmark.pdf for further details. In some jurisdictions there may be additional qualifications necessary for technologists to also operate CT and/or MR components.

### Physicist

A certified Medical Physics Expert (MPE) is responsible for quality assurance of SPECT and PET systems that are in clinical use and also for identification of possible malfunctions of these systems. The MPE is also responsible for the optimal implementation of procedures considering national and international radiation protection safety standards, both for patients and for personnel.

## Procedure/specifications of the examination

### Request/history

1. The nuclear medicine imaging facility should check with the radiopharmaceutical provider to ensure availability before scheduling the exam. Advanced notice may be required for tracer delivery.
2. The requisition should include a brief description of symptoms and the clinical question. Information should be obtained regarding the following:

- Past or current recreational drug use, head trauma, stroke, psychiatric illness, epilepsy, or tumor.
- Neurologic symptoms: type, duration, and left- or right-sidedness.
- Current medications and when last taken.
- Patient’s ability to lie still for approximately 30–45 min (for SPECT imaging with [^123^I]FP-CIT).
- Prior brain imaging studies (e.g., CT, MRI, PET, and SPECT).

### Radiopharmaceuticals

#### Presynaptic tracers

- **[**^**123**^**I]β-CIT**: 2β-carboxymethoxy-3β-(4-[^123^I]iodophenyl)tropane
- **[**^**123**^**I]FP-CIT**: N-3-fluoropropyl-2β-carbomethoxy-3β-(4-[^123^I]iodophenyl)nortropane (^[123I]^-ioflupane)
- **[**^**99m**^**Tc]TRODAT-1**: [2[[2-[[[3-(4-chlorophenyl)-8-methyl-8-azabicyclo[3,2,1]-oct-2-yl]-methyl](2-mercaptoethyl)amino]ethyl]amino]ethanethiolato(3-)-N2,N2’,S2,S2]oxo-[1R-exo-exo)])- [99mTc]-technetium
- **[**^**18**^**F]fluorodopa**: 3,4-dihydroxy-6-[^18^F]fluoro-L-phenylalanine
- **[**^**11**^**C]PE2I**: N-(3-iodopro-2*E*-enyl)-2β-carbo[^11^C]methoxy-3β-(4′-methylphenyl)nortropane
- **[**^**18**^**F]FE-PE2I**: N-(3-iodoprop-2E-enyl)-2β-carbo[^18^F]fluoroethoxy-3β-(4-methylphenyl)-nortropane
- **(+)[**^**11**^**C]dihydrotetrabenazine**: (+)2-Hydroxy-3-isobutyl-9-[^11^C]methoxy-10-methoxy-1,2,3,4,6,7,-hexahydro-11bH-bezo[α]-quinolizine; (+)[^11^C]DTBZ
- **(+)[**^**18**^**F]FP-DTBZ**: (+)-α-9-O-(3-[^18^F]fluoropropyl)DTBZ; [^18^F]AV-133

#### Postsynaptic tracers

- **[**^**123**^**I]IBZM**: (S)-3-[^123^I]iodo-N-[(1-ethyl-2-pyrrolidinyl)]methyl-2-hydroxy-6-methoxybenzamide
- **[**^**123**^**I]epidepride**: (S)-N-((1-ethyl-2-pyrrolidinyl) methyl)-5-[^123^I]iodo-2,3-dimethoxybenzamide; [^123^I]epidepride is the iodine analogue of isoremoxipride (FLB 457)
- **[**^**18**^**F]fallypride**: 5-(3-[^18^F]fluoropropyl)-2,3-dimethoxy-N-[(2S)-1-prop-2-enylpyrrolidin-2-yl]methyl]benzamide; [^18^F]N-allyl-5-fluorpropylepidepride
- **[**^**18**^**F]desmethoxyfallypride**: (S)-N-((1-allyl-2-pyrrolidinyl)methyl)-5-(3-[^18^F]fluoropropyl)-2-methoxybenzamide; [^18^F]DMFP
- **[**^**11**^**C]raclopride**: (2S)-3,5-Dichloro-N-[(1-ethyl-2-pyrrolidinyl)methyl]- 6-hydroxy-2-[^11^C]methoxybenzamide

## Procedure/specifications of the examination for presynaptic dopaminergic imaging with SPECT using ^123^I-labeled dopamine transporter ligands and with PET using [^18^F]fluorodopa

As of 2019, among SPECT tracers, only [^123^I]FP-CIT has been approved by both the US Food and Drug Administration (FDA) and the European Medicines Agency (EMA) for testing of dopaminergic neuronal integrity in suspected parkinsonian syndromes. A clinical alternative for DAT SPECT imaging is [^18^F]fluorodopa PET. [^18^F]fluorodopa is approved by EMA. Until 2019, [^18^F]fluorodopa was not approved by US FDA for marketing by manufacturer(s) nor distributing/dispensing by commercial radiopharmacies. In October 2019, a US academic medical center received FDA approval to manufacture [^18^F]fluorodopa for clinical use [29]. This approval may facilitate production and distribution of [^18^F]fluorodopa by other centers as well as commercial radiopharmacies for clinical applications. US medical centers can continue to use [^18^F]fluorodopa in human subjects under an investigational new drug application (IND), as approved by the FDA for clinical research purposes.

From the pathophysiological point of view, it should be noted that the effect of dopaminergic neurodegeneration as detected with [^18^F]fluorodopa PET tends to be smaller than with DAT SPECT [30]. Indeed, the pathophysiology of DAT and DOPA reduction in the nigrostriatal degeneration follows different timeframes as the two processes are parallel but not synchronous. This is presumably due to the presence, in the presymptomatic and early symptomatic phases, of opposite compensatory mechanisms to the reduction of the number of dopaminergic terminals: on one hand the reduction of DAT, which increases the synaptic availability of dopamine, and on the other hand, the upregulation of DOPA conversion to dopamine by aromatic L-amino-acid decarboxylase (AADC) in the nerve terminal [31]. A recent, meta-analysis of 142 positron emission tomography and single photon emission computed tomography studies demonstrated that AADC defect is consistently smaller than the dopamine transporter and vesicular monoamine transporter 2 defects, suggesting upregulation of AADC function in IPD [30, 31]. However, available studies in small number of patients and controls demonstrated that DAT SPECT and [^18^F]fluorodopa PET scans are both able to diagnose presynaptic dopaminergic deficits in early phases of PD with excellent sensitivity and specificity [32].

### SPECT using iodine-123-labeled dopamine transporter ligands

#### Patient preparation and precautions

##### Prearrival

Drugs that bind to the dopamine transporter with high affinity may interfere with DAT binding ligands. Whether or not discontinuation of these drugs prior to tracer administration may minimize the interference has not been determined by controlled in vivo or in vitro studies. The benefits and risks of stopping medication that may interfere with the reliability of the information in the images should be evaluated on an individual basis. Nevertheless, in general, it is recommended that just before the investigation, patients avoid taking any medication or drugs of abuse that could significantly affect visual and semiquantitative analysis of DAT binding ligands (except if the specific aim of the study is to assess the effect of such medication on DAT binding) [33]. See Table 1.

- Check for medications or drugs of abuse that alter tracer binding. Whenever possibile, patients should stop such medications for at least 5 half-lives.
- Cocaine, amphetamines, and methylphenidate are high-affinity DAT blockers that severely decrease tracer binding to DAT. Modafinil, used in the treatment of narcolepsy, at therapeutic doses significantly blocks dopamine transporter binding similarly to methylphenidate [34]. Indeed, while not psychoactive, cannabidiol acts as an anandamide reuptake inhibitor, which can decrease DAT function [35].
- Ephedrine and phentermine, particularly when used as tablets, some antidepressant (bupropion, radafaxine), anesthetics (ketamine, phencyclidine, and isoflurane), opioids (fentanyl), and anticholinergic drugs (benzatropine) may also decrease, to a lesser extent, DAT ligands binding
- Selective serotonin reuptake inhibitors (SSRI) may increase both [^123^I]β-CIT and [^123^I]-FP-CIT striatal binding to DAT by 20% and 10%, respectively [36]. The underlying mechanism is unknown. It has been speculated that mild [^123^I]-FP-CIT blocking of the SERT in the occipital cortex might result in a higher striatal binding ratio, although a direct interaction between the dopaminergic and serotonergic system has been also hypothesized [37]. Sertraline has a relative higher affinity for DAT that might lead to decreased striatal tracer binding. However, it has been hypothesized that these effects may be counterbalanced by the 10% increase in binding related to serotonin blocking [37]. Overall, these effects, although small (especially for [^123^I]-FP-CIT), should be considered in the framework of research studies, but they should not significantly affect the interpretation of visual assessments in clinical routine application [33].
- Chronic lithium treatment might significantly reduce DAT binding as demonstrated in a patient with clinical parkinsonism, a finding reversed after lithium withdrawal in parallel with recovery from parkinsonian symptoms. A possible mechanism could be the impaired synaptic dopamine (DA) release induced by lithium leading to compensatory downregulation of membrane DAT [37].
- Neuroleptics and cholinesterase inhibitors should not interfere significantly with tracer binding to DAT [36].
- Anti-parkinsonian drugs (e.g., L-DOPA, dopamine agonists, monoamine oxidase B inhibitors, N-methyl-D-aspartate receptor blockers, amantadine, and catechol-O-methyltransferase inhibitors in standard dosages) do not significantly interfere with [^123^I]-FP-CIT binding to DAT [36, 38].
- Nicotine use (smoking, vaping, chewing tobacco) may interfere with DAT availability [39]; however, it has been suggested that this effect is too small to affect scan interpretation.

**Table 1:** Medication and drug abuse that may significantly influence the visual and quantitative analysis of [^123^I]FP-CIT SPECT (modified from reference 11 [Darcourt et al. 2010] with permission from Springer)

Drug class	Drug name	Effect*
Cocaine		May decrease striatal [^123^I]FP-CIT binding (2 days)
Amphetamines	d-Amphetamine, methamphetamine, methylphenidate	May decrease striatal [^123^I]FP-CIT binding (7 days)
CNS stimulants	Phentermine or ephedrines	May decrease striatal [^123^I]FP-CIT binding; influences are likely when used as tablets (1 day)
Modafinil		May decrease striatal [^123^I]FP-CIT binding (3 days)
Antidepressants	Mazindol, bupropion, radafaxine	May decrease striatal [^123^I]FP-CIT binding (3 days for mazindol; 8 days for bupropion)
Adrenergic agonists	Phenylephrine or norepinephrine	May increase striatal [^123^I]FP-CIT binding; influences are likely when infused at high doses
Anticholinergic drugs		Benztropine may decrease striatal binding ratios; other anticholinergics may increase these ratios, which will likely not affect visual assessments
Opioids	Fentanyl	May decrease striatal [^123^I]FP-CIT binding (5 days)
Anesthetics	Ketamine, PCP, isoflurane	May decrease striatal [^123^I]FP-CIT binding; of interest particularly for animal SPECT studies, although ketamine and PCP are sometimes used illicitly (1 day)

*In brackets, time interval approximately equal to 5 half-lives

A more detailed discussion of drug effects on DAT SPECT can be found in reference 33.

##### Preinjection

To reduce ^123^I radiation exposure to the thyroid, a single administration of potassium iodide oral solution or Lugol’s solution (equivalent to 100 mg iodide) or potassium iodide tablet, potassium perchlorate (400 mg), or sodium perchlorate (600 mg) should be given at least 1 h before injection of [^123^I]-FP-CIT (unless known sensitivities to these products are reported). Even in the absence of a blocking agent, the radiation dose to the thyroid would be low [13]. Injection of the tracers is contraindicated in case of known hypersensitivity to the active substance or to any of its excipients. An iodine allergy is, however, not a contraindication to receiving this tracer. Anti-allergy premedication can be considered in specific cases.

##### Precautions

Continuous supervision of the patients during images acquisition is mandatory. This is especially important for uncooperative subjects due to cognitive/behavioral symptoms, which can occur in presence of some parkinsonian syndromes.

##### Preparation of the radiopharmaceutical

Radiopharmaceuticals is delivered ready to use.

##### Quality control check

Quality control parameters as listed in the package inserts. The instructions provided by the manufacturer should be followed.

#### Injection

Injection of the radiopharmaceutical should be performed (within the time frame given by the manufacturer) as a slow bolus over followed by a saline flush of the intravenous line.

##### Administered dosage

1. Adults: 110–250 MBq (typically 185 MBq) for both [^123^I]FP-CIT and [^123^I]β-CIT
2. Children: Currently, there are no established clinical indications and safety and efficacy have not been established in pediatric patients. Accordingly, its If indicated in the future, activity should be administered according to the recommendations of the EANM Pediatric Task Group.
3. The effect of renal or hepatic impairment on DAT SPECT imaging has not been established. Because [^123^I]-FP-CIT is excreted by the kidneys, patients with severe renal impairment may have increased radiation exposure and altered DAT SPECT images. The patient should also be encouraged to stay hydrated and void frequently the remainder of the day to minimize radiation exposure to the bladder [14]. Hypersensitivity and reactions in the site of injection have been described. The most common adverse reactions as reported in clinical trials were nausea, headache, vertigo, and dizziness, which occurred in less than 1% of subjects.

#### Equipment specifications

1. Multiple detector (triple or dual head) or other dedicated SPECT cameras for brain imaging should be used for data acquisition [40, 41]. Single detector units are not recommended as excessively long scan times is needed to acquire the required number of counts. They may be used only if the scan time is prolonged appropriately; injected activity in the upper permissible range is used to produce high-quality images.
2. Low energy high (or ultrahigh) resolution (LEHR or LEUHR) parallel-hole collimators are the most frequently available collimator sets for brain imaging. All-purpose collimators are not suitable, while fan-beam collimators have a more advantageous trade-off between resolution and count rate capability with respect to parallel-hole collimators. Medium energy collimators have a lower spatial resolution although they have advantages due to septal penetration [42]. If available, collimator sets specifically adapted to the characteristics of ^123^I may be used. [42]

#### Acquisition protocol

##### Timing

1. SPECT should be started when the ratio of striatal-to-occipital tracer binding is stable [21]. In fact, waiting for stability of the ratio guarantees the most reliable data from a semiquantitative point of view and is optimal for visual assessment when low background interference is needed.
  - [^123^I]FP-CIT: 3 to 6 h postinjection
  - [^123^I]β-CIT: 18 to 24 h postinjection
2. Each center should maintain a standardized postinjection imaging time (e.g., always start imaging at 3 h post radiotracer injection for [^123^I]FP-CIT) to favor comparability of data between subjects and intraindividual follow-up studies.
3. Since tracer binding is not determined by flow effects, patients do not need to stay in a quiet/dim environment. There are no dietary restrictions, pre- or postinjection.
4. If the patient is claustrophobic, or is unable to lie still, mild sedation with a short-acting benzodiazepine may be given prior to imaging and will not affect scan quality or quantification. In this circumstance, the patient should not operate machines or drive a car for the rest of the day

##### Positioning

1. The patient should void immediately prior to imaging.
2. The patient should lie on the couch in a supine position with the head resting in the head holder in a comfortable orientation. A light head restraint may be considered to reduce the risk of motion artifacts.
3. Although proper alignment of the head would be preferable, patient comfort should be favorite to prevent movement during acquisition of the images. Tilt and orientation can be adjusted during postprocessing.
4. The arms should be supported at the side or in front of the patient. Additional leg support may be used to improve comfort. Binding the shoulders may prevent movement and may allow to reduce the rotational radius of the heads of the camera.
5. The striatum and occipital lobes must be in the field of view. The inclusion of cerebellum might be omitted in the acquisition to optimize radius of rotation and spatial resolution only in patients with challenging positioning of the head [43]. It should be noted, however, that when the cerebellum is not in the field of view, reorientation accuracy and spatial registration might be affected (see below).

##### Acquisition parameters

1. Rotational radius: it should be typically 11–15 cm (as small as possible with appropriate patient protection.
2. Photopeak: 159 KeV ± 10% or 159 KeV ± 7.5%, as recommended by manufacturer. Additional energy windows for scatter correction may be used as needed.
3. Mode: step and shoot mode is used predominantly. Continuous mode acquisition may provide shorter total scan times and reduce mechanical wear to the system
4. Matrix: at least 128 × 128
5. Zoom: adjust zoom factor to achieve a pixel size of 2.5–4.5 mm. In general acquisition, pixel size should be one-third to one-half of the expected resolution.
6. Rotation: 360° coverage
7. Angular step: ~ 3° increments (for step and shoot mode).
8. Frame time: the number of seconds per position depends on the sensitivity of the system, but usually 25–40 s are required.
9. Total detected events in the photopeak window: should be > 1.5 million total counts for [^123^I]-FP-CIT; > 1 million for [^123^I]β-CIT. In this framework, it is helpful, prior to start images acquisition, to use lateral or anterior projections to ensure that the count rate is appropriate to achieve the required number of counts.
10. SPECT/CT: the SPECT image acquisition on SPECT/CT systems should follow the general recommendations for DAT SPECT image acquisition.
11. CT-based attenuation correction (CTAC) may be performed with SPECT/CT systems following manufacturer’s protocol. Overall in the few available studies comparing data obtained using CTAC with data obtained using uniform AC, minor differences were observed, without impact on diagnostic performance. It should be noted that when SPECT/CT devices are available, CT is generally used for attenuation correction and anatomical localization only. Accordingly, the CT acquisition mode should use the protocol that delivers the lowest possible radiation dose to the subject (e.g., a relatively low dose protocol) that retains the quantitative accuracy of corrections for attenuation and anatomic localization. According to Guidelines of the Quantitative Imaging Biomarkers Alliance of the Radiological Society of North America (QIBA-RSNA), when CT is used for attenuation correction only, the CT can be performed with 5–10 mAs. [44].
12. Segmentation of data acquisition into multiple sequential acquisitions allow to remove segments of projection data with patient motion thus reducing artifacts.

#### Image processing

##### Review of projection data

1. Images should be checked prior to reconstruction. It is difficult to recognize movement in the reconstructed SPECT slices. Accordingly, the raw projection images should be watched in cine mode or in sinogram mode.
2. Given the 8–10 mm spatial resolution of SPECT, small amounts of motion may be tolerated. For more significant motion, repeated scanning is recommended.

##### Image reconstruction

1. Methods: filtered back projection (FBP) or iterative reconstruction. In general, FBP results appears to be more consistent across vendors. However, iterative reconstruction may be preferred to help reduce streak artifact and for more robust physical correction algorithms.
2. If iterative reconstruction is used, typically, about 100 EM equivalent iterations (iterations × subsets) should be used, e.g., 10 iterations and 10 subsets for 120 projections or 12 iterations and 8 subsets for 128 projections [45]. However, if normal databases for semiquantitative assessment are used, given that the choice of iterations and subsets will affect semiquantitative values, chosen reconstruction parameters should match those used in the normal databases.
3. Ideally the entire brain volume should be reconstructed to assist in the reformatting of data. Semiquantitative software may, in fact, require the cerebellum as a reference region or the inclusion of the cerebellum may be important in the spatial registration process.

##### Filtering

1. Data should be filtered in all three dimensions (x, y, z) by two-dimensional prefiltering of the projection data. A three-dimensional postfilter to the reconstructed data can also be applied. The second option is recommended for iterative methods because of the underlying assumption of Poisson distribution of the detected counts.
2. Low-pass (e.g., Butterworth or Gaussian) filters are suitable to reduce image noise, but not at the detriment of spatial resolution. Caution should be used if spatially varying filters area applied as they can results in artifacts.
3. If resolution modeling is incorporated into the reconstruction, the noise suppression introduced in the algorithm may remove or reduce the need for additional filtering.

##### Corrections

1. The use of corrections (attenuation, scatter and septal penetration, referred in the following simply as “scatter correction”, and partial volume effect) do not necessarily benefit visual interpretation although it will significantly affect the values derived from semiquantitative analysis. Corrections may be considered to reduce quantitative bias, and, when accuracy is desired, all three corrections should be applied [46, 47].
2. If normal databases are to be used, the reconstruction should match that used to create the normal database.
3. Chang attenuation correction with a uniform attenuation map can be used effectively with an appropriate linear attenuation correction coefficient for ^123^I and careful contouring of the head/scalp. Typically, the linear attenuation factor should be in the range μ = 0.10–0.12 cm^−1^ without scatter correction, while the μ value in water of 0.143 cm^−1^ should be used with scatter correction [9, 11, 12].
4. CT-based attenuation correction is more robust and can account for head holder attenuation. Obviously, the choice of low-dose “attenuation correction only” CT versus high-dose “attenuation correction and diagnostic” CT has important implications for the radiation dose, and a full diagnostic CT should be used only when justified by the clinical indication.
5. Scatter (and septal penetration) correction can improve visual contrast and reduce bias in semiquantitative analysis. It can be performed using either energy-window based corrections [48] or transmission-dependent convolution subtraction [49]. The former is simple to implement and can account for extra-cerebral activity but increases image noise and therefore worsens quantitative precision. The latter handles noise better but is unable to account for septal penetration of ^123^I high energy gammas, which can vary significantly across scanners [50]. Modern vendor software allows for the application of scatter and attenuation correction and adherence to manufacturer-specified parameters is recommended.
6. Due to the limited spatial resolution of SPECT systems, imaging small structures in the basal ganglia suffers from large partial volume effects (PVE), which causes the most significant bias/loss in signal during semiquantitative analysis [51]. Resolution modeling techniques in the reconstruction can compensate for some of these losses, although overshoot artifacts associated with the Gibbs phenomena [52] mean that such techniques should be used with caution. It should also be noted that for some commercial systems, application of resolution modeling is combined with other corrections and cannot be decoupled.

#### Quality control

##### Prerequisites

1. All SPECT systems should be subjected to a quality control program (AAPM (In press), EANM 2010, IAEA 2009) [53, 54]
2. For SPECT/CT systems, CT sub-systems should also be under a quality control program (IAEA 2012) and have additional SPECT/CT quality control (QC) tests performed [54]
3. The SPECT system must be set up and calibrated for imaging ^123^I.
4. Given the small structures in the basal ganglia, reconstructed SPECT spatial resolution should be ≤ 10 mm (FWHM).
5. If using Chang attenuation correction, ^123^I SPECT uniformity using the reconstruction and corrections for clinical imaging should be checked to ensure that an appropriate linear attenuation factor is being used.
6. It can be helpful to image an anthropomorphic striatal phantom to assess SPECT image quality using clinical acquisition and reconstruction parameters.

##### Periodic QC

1. Intrinsic (without the collimator) uniformity to ^123^I should be checked.
2. Center of rotation checks with the collimators used for DAT SPECT should be assessed.
3. If using SPECT/CT systems, the alignment/registration of SPECT and CT should be checked to ensure good localization and attenuation correction.

##### QC on the day of DAT SPECT

1. Uniformity (intrinsic or system) of detector response should be checked daily prior to patient injection.
2. The collimators should be checked for mechanical damage. A high-count extrinsic uniformity test should be performed if collimators damage is suspected.
3. The peaking of the camera to ^123^I should be checked with the patient on the scanner.

#### Interpretation/semiquantification

##### Reformatted sections and reorientation

Transaxial slices should be reformatted into three orthogonal planes for visual assessment. Generate transverse sections parallel to a given anatomic orientation (e.g., anterior commissural to posterior commissural line; AC-PC) ensuring a high degree of standardization in plane orientation. Create coronal and sagittal sections orthogonal to the transverse sections.

##### Visual interpretation

Patients become symptomatic after a significant number of striatal synapses have degenerated. Accordingly, visual interpretation of images is usually sufficient for clinical report in the majority of cases. Accurate reporting with trained readers has been demonstrated using visual interpretation only [15–18, 55, 56].

1. Readers are recommended to select one color scale for consistency. Level of contrast and background subtraction should be carefully checked as inappropriate thresholding may result in artifacts.
2. Data evaluation should consider age and relevant morphological information (CT, MRI). Structural lesions in the basal ganglia and midbrain should be considered with great attention especially when present in structures selected as reference region for semiquantitative evaluation. Review of previous brain CT and MRI is mandatory. In fact, anatomic lesions and central atrophy may alter the tracer binding, location, or shape of the striatal structures.
3. Visual assessment is usually sufficient for evaluating striatal left/right symmetry and striatal subregions.
4. On transaxial images, the normal striata should look as comma-shaped with symmetric well-delineated borders (although mild asymmetry may be present in also in normal controls).
5. Care should be taken in the alignment of the head, particularly in the coronal plane, in order to avoid artifactual left-right asymmetry, which may lead to visual misinterpretation of the scan. It is recommended, therefore, to visualize all the transaxial slices containing striatal signal so that real asymmetry can be differentiated from an insufficiently corrected head tilt
6. In abnormal scans, striata present reduced intensity on one or both sides, with oval or circular shape. The level of striatal activity should be compared with the background activity.
7. Common patterns for abnormalities: in IPD, the disease first becomes visible in the posterior putamen contralateral to the neurologic signs [55, 56]. In fact, in early IPD, the putamen is usually more markedly affected than the caudate nucleus. Furthermore, the putamen of the less affected hemisphere is also early involved (often before the caudate nucleus of the most affected side).This pattern results in the so-called dot shape (as opposed to the normal comma shape) typical of IPD in the earliest stages of disease.
8. The presence of a dot shape with uptake reduction in both putamina has been repeatedly described in both patients with hemiparkinsonism and even in patients with premotor PD (i.e., in clinical trials carried out in patients with anosmia and/or REM sleep behavior disorders [24, 25, 57, 58]. These findings testify to the high sensitivity of DAT SPECT in the earliest stages of disease and its high negative predictive value, which still remains one of its main strengths.
9. In IPD, the DAT binding is often first reduced in the putamen contralateral to the side of the body showing the most severe signs of disease, then the reduction in tracer uptake progresses to the contralateral putamen and anteriorly and ipsilaterally to the caudate over time. Correlation of scores/scales measuring severity of motor impairment and disability (UPDRIII and Hoehn and Yahr Scale) on one side and degree of DAT binding as assessed by DAT SPECT on the other have been demonstrated in early IPD [59].However, later on in the course of the disease, the striatal DAT measures reach a floor effect and correlation with ongoing disease progression is lost [60]. In any case, IPD usually is characterized by reduced uptake in all the basal ganglia structures only in advanced stages of disease, and the presence of strong decrease in signal-to-noise ratio in patients with very mild motor symptoms might be due to tracer extravasation.
10. In DLB, early involvement of the caudate nucleus may cause the difference in tracer uptake between the putamen and the caudate to be less apparent than in PD. This may result in images with “weak commas” or “balanced loss” [52]. In general, atypical parkinsonian syndromes tend to have a more symmetrically reduced uptake. The uptake of the caudatus is also reduced relatively earlier with respect to IPD. However, there is too much overlap between the disease patterns to allow for adequate discrimination between PD/DLB, MSA, PSP, and CBD on an individual level [18, 52].
11. Since late 90s, some patients (10% to 15%) that were enrolled in PD clinical trials as having newly diagnosed PD demonstrated normal DAT SPECT and were originally defined as subjects having a scan without evidence of dopaminergic deficit (SWEDD) [61]. The concept of SWEDD is, however, highly debated and controversial and represents a heterogeneous entity presumably due to misdiagnosis and/or SPECT misinterpretation. Longitudinal assessment demonstrated that subjects with a SWEDD at baseline continue to have a SWEDD in follow-up scans. In fact, it was more recently demonstrated that they have minimal evidence of clinical or imaging PD progression. This new evidence strongly suggests that SWEDD subjects are unlikely to have IPD [52, 61].
12. DAT ligand binding is normal in essential tremor, drug-induced parkinsonism, and psychogenic parkinsonism. This difference allow an accurate differential diagnosis with respect to neurodegenerative parkinsonian syndromes [22, 23, 56].
13. The term vascular parkinsonism is controversial, and ill-defined. Basal ganglia vascular lesions are very common and will cause parkinsonism in only a minority of patients, and neither infarct site nor the size can predict the clinical presentation. In the literature, DAT ligand binding has been described as normal or only slightly diminished, except when an infarct directly involves a striatal structure. Even then, a deficit from an infarct often gives a “punched-out” appearance. The pattern has a different aspect with respect to the appearance of abnormal DAT SPECT in typical presynaptic parkinsonian syndrome deficit. Review of a recent MRI scan can be of help in this type of patients [62–64]. However, an infarct placed adjacently to striatal structures may give a more diffuse reduction through the PVE mimicking early IPD. It should be noted that vascular lesions in or near the basal ganglia are common in the typical age group scanned and is a rare cause of parkinsonism. Enlargement of perivascular spaces (Virchow–Robin spaces) are generally most prominent in the inferior basal ganglia and may lead to a diffuse reduction in striatal uptake following the distribution of spaces [65]. As “incidental” cerebrovascular lesions are found in 20–30% of patients with histologically confirmed IPD, the basal ganglia structural lesions are not uncommonly combined with a true nigrostriatal dysfunction [66].
14. DAT SPECT is an accurate way to differentiates between Alzheimer’s disease (AD) and DLB [20]. In fact, striatal binding is usually normal or only mildly diminished in AD and is significantly decreased in DLB [19, 20]. In particular, given its high specificity (20% higher with respect to clinical diagnosis), the revised criteria for the diagnosis of DLB further reinforced the role of DAT SPECT considering it as a diagnostic biomarker [20]. It should be noted, however, that approximately 10% of patients who met pathologic criteria for DLB can show a normal DAT SPECT at the time of the clinical diagnosis (possibly becoming abnormal after 1.5–2 years) [67, 68]. In a recent autopsy study, this finding was related to topographical heterogeneity of α-synuclein deposition [19]. In fact, this subgroup of patients was characterized by only mild cell loss and α-synuclein pathology of low severity in the substantia nigra and moderate severity at the cortical level.
15. Consistent with the clinical and neuroradiological asymmetry characterizing CBD, a more asymmetric reduced uptake (involving both putamen and caudate on the hemisphere contralateral to the most affected body side) has been reported in patients with CBD [69]. DAT SPECT is not sufficiently accurate to support the differential diagnosis between IPD and CBD. However, a markedly asymmetrical pattern of DAT binding reduction should be specifically emphasized in the final report if the clinical suspicion is CBD in order to guide the subsequent diagnostic workout (i.e., ^123^I-mIBG and [^18^F]FDG PET might be useful in this scenario) [70, 71]. Unexpectedly, normal DAT SPECT findings might also occur in patients with clinical corticobasal syndrome (CBS). This finding is likely to be related to an underlying etiology different from CBD (AD, frontotemporal dementia) [72]. However, this issue needs further clarification, which might be provided by further studies based on multimodal biomarker (CSF, amyloid PET, and possibly TAU PET) in patients with CBS/CBD.
16. DAT SPECT is abnormal in 30–60% of patients with sporadic frontotemporal dementia (FTD), but usually less pronounced than in DLB/IPD [72–75]. However, some cases with marked abnormal DAT SPECT have been reported in specific subtypes of FTD (i.e., familial FTD with parkinsonism linked to chromosome 17q—FTDP-17) [72–75]. DAT SPECT is not a direct marker of α-synuclein pathology but only a measure of its effect on nigrostriatal neurons, which can be damaged by other pathology [76].

A schematic summary of expected DAT SPECT findings in the most frequent clinical entities included in the differential diagnosis of parkinsonism is provided in Table 2.

**Table 2:** Overview of DAT SPECT* findings in patients with parkinsonism

Disease	Findings	References
Idiopathic Parkinson’s disease	A *dot shape* is typical since the earliest (and premotor) stages of disease. The putamen (in particular the posterior part) of the most affected hemisphere is more severely affected than the caudate nucleus the putamen of the less affected hemisphere tends to be early involved (often before the caudate nucleus of the most affected side)	[15, 55–58]
Essential tremor Psychogenic parkinsonism Drug-induced parkinsonism Alzheimer’s disease	Normal *comma shape*	[22, 23, 55]
Vascular parkinsonism	Poorly defined clinical condition. In patients with small vascular lesions in the basal ganglia, DAT tracer uptake is normal or only slightly diminished. Definite vascular parkinsonism should include a documented ischemic or hemorrhagic lesions in the substantia nigra or nigrostriatal pathway leading to striatal presynaptic DAT deficiency (see main text).	[62–66]
Dementia with Lewy body	Reduced uptake substantially overlapping IPD features	[18–20, 59, 67, 68]
	Around 10% of patients with pathologic proven DLB show a normal DAT SPECT at the time of the clinical diagnosis (possibly becoming abnormal after 1.5–2 years)	
Multiple system atrophy	Reduced uptake, often overlapping IPD features (uptake reduction tend to be more symmetric as compared with IPD)	[60]
Progressive supranuclear palsy	Reduced uptake, often overlapping IPD features (uptake reduction tend to be more symmetric and to involve the caudate nucleus earlier in disease course as compared with IPD)	[60]
Corticobasal degeneration	Reduced uptake, overlapping IPD features (uptake reduction sometimes more asymmetric as compared with IPD and often involving both putamen and caudate head)	[69–72]
Frontotemporal dementia	Reduced uptake in 30–60% of patients with sporadic FTD (usually less pronounced than in IPD)	[72–76]

*Given the greater number of available studies describing normal and abnormal distribution of DAT SPECT with respect to [^18^F]fluorodopa and the different pathophysiology and timeframes of DAT and DOPA reduction and considering the different resolution of PET and SPECT, we have limited the present summary to DAT SPECT data. However, it should be noted that normal distribution and the hereby described abnormal patterns in different parkinsonian syndromes are expected to be very similar for DAT SPECT and [^18^F]fluorodopa PET (see main text for further details)

##### Semiquantification

The output images from image reconstruction are considered the input for image analysis.

1. In the clinical setting, semiquantitative analysis can be an adjunct to visual interpretation. Some studies have shown the addition of quantitative information results in improved diagnostic performance [50, 77, 78]. In research settings, semiquantitative methods are considered to provide a more precise assessment of dopamine transporter density, allowing for more reproducible measurements of disease and response to therapy.
2. The most commonly used semiquantitative outcome measure in both research and clinical settings is the specific binding ratio (SBR) calculated as the striatal target-to-background ratio [79, 80]. SBR is related to, but not a specific measure of, the density of dopamine transporters; however, it is not a direct measure of DAT density (Bmax), synaptic density, or neuronal number. Many factors that are unrelated to the density of dopamine transporters influence the determination of SBR (Tables 3 reports both biological and technical factors affecting SBR).
3. It should be noted that interobserver variation and errors can be considerable during the placement of the regions of interest (ROIs) for semiquantification. Variability in the reorientation of the brain can also affect the interpretation.
4. Semiquantification allow more objective measurements of SBR (with improved inter-reader agreement and reader confidence). The potential correlation between clinical variables and semiquantitative parameters expressing loss of presynaptic dopaminergic neurons is a further added value [81].
5. ROI-based techniques can be used to assess specific DAT binding in the striatum and striatal subregions. Transverse slices are generally selected and the slices with the highest striatal uptake or the entire striatal volume can be considered to draw manual ROIs. The use of standardized ROIs is helpful in limiting operator variability to the positioning task. The shape of the template ROIs could be either geometrical or anatomical, the size should be at least twice the FWHM.
  Reference regions with absent (or low) DAT density are used to assess nonspecific binding. The reference region is ideally the cerebellum as it contains no known dopaminergic neurons. The occipital cortex can be also used particularly when the axial field of view is limited. The methodology used for defining the reference region should be consistent across all patients measured. It is particularly important when comparing measurements at different time points in the same patient
  If anatomical scans (MRI or CT) are available, volumetric regions encompassing the anatomic extent of the basal ganglia can be used. This is particularly important when low specific binding is expected (e.g., in case of a severe loss or blockade of the DAT), especially in the absence of an automated method.
6. Once the volumetric ROI (VOI) are placed, SBR values are generally obtained as follows:

**Table 3:** Factors affecting specific binding ratio (SBR) in [^123^I]-FP-CIT SPECT imaging

**Biological factors**	
Dopamine transporter density	
Age and gender	
Pharmacokinetics factors (rate of uptake, metabolism, and elimination of tracers)	
Genetic: allelic variants of DAT	
Drugs competing with the tracer for DAT binding	
**Technical factors**	
Patient ability to remain motionless in the camera	
Equipment	
Resolution and sensitivity of selected camera	
Collimator	
CT-based vs. uniform attenuation map*	
Performance drifts overtime	
Acquisition	
Dose extravasations (counts in image**)	
Time postinjection	
Head position^§^	
Patient movement	
Radius of rotation	
Reconstruction	
Osem vs. FBP	
Filtration	
Attenuation correction	
Scatter and septal penetration correction	
PVE correction	
Choice of SBR algorithm^§§^	
Size and placement (spatial registration) of regions of interest (including background region used)	

*PVE* partial volume effect, *CT* computed tomography

*Relevant in case of ill-defined attenuation map boundaries, significant bed or head holder attenuation, or incorrect mu value

^**^Counts affect SBR at very low count levels (< 1 million) as the regions of interest (ROI) provide an average signal (unless too small ROIs are used)

^§^Inclusion of the whole striata and, possibly, of the whole brain in the field of view, in accordance with the requirements of the SBR algorithm on striatal and reference regions and spatial registration process

^§§^Based on direct measurements of counts concentration or on total counts in striatal VOIs

(Mean Counts of striatal VOI − Mean Counts of background VOI)/(Mean Counts of the background VOI)

- Automated semiquantification

Several approaches have been proposed to perform semiquantification of DAT SPECT, and several commercial and freeware software are available.

Some of them include a one- or two-step–based fully automated registration of the patient SPECT scan to a template or to an averaged, spatially normalized brain volume. Predefined VOIs are then automatically placed on the striatal and the background regions [82–88].

VOIs can also be defined for striatal substructures, allowing to quantify the SBR for caudate and putamen, as well as other parameters such as asymmetry between left and right putamen and caudate, putamen-to-caudate ratio (PCR), and caudate-putamen ratio (CPR).

The availability of these parameters can be useful in complex or borderline cases. In particular, PCR is most sensitive in borderline and early stage cases because of a higher degree of independence on camera, reconstruction algorithm, and background region. Some studies have reported a mild reduction of PCR with age, but there are conflicting data in the literature on this topic (possibly due to the number and age range of included subjects and the modalities of VOI drawing) [87]. Evaluation of asymmetry between left and right sides is also useful in the earliest stages, although mild asymmetry between striata or striatal subregions may occur in normal subjects [88].

All the numeric parameters are dependent on acquisition and reconstruction parameters and on the corrections applied [49, 50]. As a result, there are no universal (age-dependent) cutoffs for normal vs. abnormal [89–91]. Accordingly, each site would ideally need to establish its own reference using a healthy control group. If a local normal subject database is not available, calibration of the procedure with the characteristics of normal controls databases is needed (see below).

Differences in SBR also occur as the result of VOI strategy, with two approaches commonly employed. The first incorporates regions for the caudate and putamen on left and right sides, which tries to capture the anatomic bounds of these structures. The second approach utilizes small regions of interest sampling the tracer uptake in the caudate, mid-putamen, and posterior putamen on the right and left sides [92, 93].

A different approach for determining the SBR is used in the so-called Southampton method [93], based on the measurement of total counts from each striatum. It uses striatal VOIs of geometrical shape, sufficiently large to capture all counts originating from the striatum including those detected outside its anatomical boundaries, and a background region derived from the whole brain minus the striatal VOIs. The SBR is then calculated as follows:

Vol_StrVOI_/Vol_Str_ × (Counts_StrVOI_/counts per voxel_bkg_ × Vol_StrVOI_ − 1)

Individual computed tomography (CT) or MRI-guided methods [94] and, more recently, machine-learning techniques for pattern recognition as well as parametrization of textural patterns have also been proposed as user-independent methods for DAT SPECT result classification. These methods still remain in the realm of promising research [95, 96].

Some studies have applied PVE in the process of binding computation of the caudate nucleus, putamen, and background, but the added value of PVE in this settings has not been systematically investigated and is not recommended in routine clinical practice.

- Available healthy subjects’ database

As previously mentioned, great caution must be exercised when using SBR data from the literature as differences regarding to the camera system how the images were acquired, processed, and analyzed will result in different values [50, 87, 97, 98].

Calibrations across cameras have been described using anthropomorphic phantoms to develop relative correction factors for standardization between instruments [46, 87, 99]. The QIBA of the RSNA is developing a protocol for quantitative standardization of [^123^I]-FP-CIT SPECT analysis, which will detail a validated protocol for imaging centers to apply in order to obtain the high reproducibility [45].

In recent years, two normative database have been provided:

The ENC-DAT (European Normal Control Database of DaTSCAN) database was developed from 2009. The study was promoted by the EANM Neuroimaging Committee, in the framework of a cooperative effort among European Nuclear Medicine centers. This multicenter project aimed to collect a large number of [^123^I]FP-CIT SPECT scans of healthy controls, thus providing reference images and values of DAT availability measures across a wide age range of both genders. The final database included SPECT data from 139 healthy controls (74 men, 65 women; age range 20–83 years, mean 53 years) acquired in 13 different centers [100].

The second database of healthy controls was collected in the framework of the Parkinson’s Progression Marker Initiative (PPMI), which is a multicenter, international, longitudinal study evaluating clinical, biochemical, and imaging measures of PD progression. PPMI aimed (1) to confirm the presence or absence of a DAT deficit for PD and healthy volunteers enrolled in the study, (2) to acquire DAT SPECT data with rigorous standardized acquisition protocols, and (3) to process images through a central core lab reconstruction of raw projection data for subsequent uniform analyses to be made available to the investigator community. PPMI includes 423 progressing Parkinson’s patients and 196 age-matched controls studied over 3 to 4 years with serial [^123^I]-FP-CIT SPECT imaging and other clinical, imaging, and fluid biomarkers [101].

Normative data provided by these two multicenter initiatives demonstrated that normal aging is associated with about 5.5–6% signal loss per decade (0.6%/year). Gender had also an effect on SBR in the caudate and putamen (often not considered in the software available for comparison) [81, 94, 96]. Dependency of DAT on BMI, handedness, circadian rhythm, or season was not demonstrated [87, 100, 102].

SPECT/CT: Very few studies tested the use of CT images acquired on the SPECT/CT system to derive either warping parameters for VOI or for regional SBR assessment, and the anatomical information provided by CT did not demonstrate a relevant impact on diagnostic performance [103].

- Longitudinal data

Serial imaging within the individual patient in order to track disease progression by visual or semiquantitative analysis might be clinically useful only once it has been demonstrated that signal size is able to capture the slow progression of the disease process.

Besides providing a healthy subjects database, the PPMI initiative has provided an assessment of within-subject changes of SPECT imaging in a PD patient cohort followed longitudinally over 3–5 years. The aim is to understand the utility of DAT SPECT as a putative biomarker of PD progression after early motor signs of PD appear [104]. In fact, if intraindividual comparison is performed (i.e., baseline vs. follow-up for therapy control or assessment of disease progression), more subtle changes might need to be highlighted.

In the PPMI study, regional SBR were measured in ipsilateral and contralateral caudate, anterior putamen, and posterior putamen at baseline and 1, 2, and 4 years during the follow up of IPD patients. During the 4 years, there was a significant longitudinal change in dopamine transporter binding in all striatal regions [104]. Available analyses seem to suggest that, given the size of signal change in an early PD cohort, [^123^I]FP-CIT SPECT might be a suitable biomarker of PD progression. In fact, initial longitudinal data suggest SBR reductions over 1 year are approximately 20 times the rate of signal loss seen in normal aging [104].

#### Multicenter trial harmonization

In order to harmonize imaging equipment sites for multicenter research or trials, there are several areas to be considered [105].

1. Data acquisition: the acquisition protocol should be harmonized as much as possible. Collimators, energy windows, pixel sizes, and required number of counts are key considerations. During this process, attention should be given to manufacturer specific requirements, e.g., energy windows for scatter correction.
2. Image reconstruction and filtering: the choice of reconstruction and filtering is very important in harmonization particularly given that semiquantification is strongly dependent on the approach taken and the corrections included. Variability in reconstruction algorithms and functionality across centers may drive the choice of reconstruction to an approach that is comparable and achievable by centers, i.e., typically one with no corrections. Alternatively, to overcome this variability, reconstruction at a central core lab using a generic algorithm could be considered.
3. Comparability of system performance: the use of an anthropomorphic striatal phantom can help assess comparability of visual and semiquantitative performance across sites/scanners and is, therefore, recommended [46]. One or more data acquisitions with varying filling ratios is suggested to determine the relationship between scanner semiquantitative performances over a range of SBRs.

### Presynaptic dopaminergic imaging with PET using [^18^F]fluorodopa

#### Preparation of the radiopharmaceuticals

[^18^F]fluorodopa as all radiopharmaceuticals must be produced by qualified personnel according to cGMP or using GMP-compliant methods that conform to regulatory requirements. The quality control is carried out by the manufacturer prior to delivery of the final product when the radiopharmaceutical is delivered ready to use.

#### Patient preparation and precautions

##### Prearrival

1. Anti-parkinsonian drugs (L-dihydroxyphenylalanine (L-DOPA), dopamine agonists, monoamine oxidase B inhibitors, N-methyl-D-aspartate receptor blockers, and amantadine) do not significantly interfere with visual interpretation. However, a discontinuation of L-DOPA by at least 12 h should be considered as it has been recommended by manufacturers and applied in published studies to improve image quality (although it should not interfere visual interpretation significantly). Neuroleptics have a limited effect on [^18^F]fluorodopa kinetics and do not need to be discontinued [106].
2. Food intake affects [^18^F]fluorodopa kinetics since dietary large neutral amino acids compete with [^18^F]fluorodopa for transfer by a common carrier through the blood brain barrier [107]. Therefore, it is recommended that patients should not intake any amino acid–containing foods 4 h prior to the procedure.
3. Smoking probably does not interfere significantly with fluorodopa kinetics (although dopamine synthesis capacity seems to be increased in heavy smokers) [108].
4. Recent use of recreational drugs that directly act on the dopaminergic system (cocaine, amphetamine) should be noted.

##### Preinjection

1. Prehydration is important to reduce the radiation exposure of [^18^F]fluorodopa by stimulating bladder voiding. Patients should be encouraged to drink sufficient amounts of water and to empty their bladder prior to and after the PET examination.
2. Pretreatment with the peripheral dopa decarboxylase inhibitor carbidopa (150 mg or 2 mg/kg up to 150 mg, orally) or catechol-O-methyltransferase (COMT) inhibitor entacapone (200 mg, orally) 60–90 min prior to [^18^F]fluorodopa injection is a common practice that increases the availability of DOPA to the brain and reduces absorbed dose to the bladder and kidneys [109–111].

#### Injection

The recommended injected activity for brain imaging in adults for PET-CT is 185 MBq. If no carbidopa is given ahead of [^18^F]fluorodopa, higher doses may be necessary. [^18^F]fluorodopa should be injected as a bolus.

#### Image acquisition

##### Positioning

The patient should be in supine position, with the head in a dedicated holder and arms along the body. Ensure to have the whole brain (including the cerebellum) in the field of view. Extreme neck extension or flexion should be avoided.

1. Head stability
  To avoid head movements during the examination, the patient should be informed immediately before starting the acquisition (the same precautions described for DAT SPECT images acquisition should be used).

##### Acquisition protocol for PET/CT systems [112, 113]

1. The preferred sequence of PET imaging is:

CT scout topogram for PET(/CT) to setup field of view.

For attenuation correction: low-dose CT scan (see before paragraphs on SPECT/CT) or transmission scan. If a PET/MRI system is used, an MR-AC scan for MR-based attenuation correction should be acquired. Static or dynamic single field of view (FOV) PET acquisition

2. Image acquisition should be performed in 3D data acquisition mode, and attenuation correction should be based on low-dose CT or on a 511 keV transmission scan. If 511 keV transmission scanning is used, the transmission images should be acquired before tracer injection. CT parameters should always be chosen to ensure the lowest radiation exposure to the patient that is compatible with this purpose.
3. A fixed time for the start of image acquisition should be taken.
4. Static acquisition: 10–20 min static image acquisition obtained 70–90 min postinjection is recommended [114, 115]. If movement artifacts are expected, it can be helpful to acquire the static time window dynamically, e.g., in 5-min frames, or in list-mode, check the sinograms, and use only the sinograms of the properly acquired motion-free time period for reconstruction.
5. Dynamic or list-mode acquisition [115, 116]: a protocol of at least 90 min acquisition is required for the calculation of the [18F]fluorodopa influx constant (K_i_). The acquisition starts at the time of injection using short 30-s time frames that progressively increase to 5 min in duration. During the first 10-min postinjection, the following image acquisition frames can be used: 6 frames of 30 s and 2 frames of 210 s allowing to precisely obtain information of the tracer uptake phase. During the remaining 80 min, frames of 5 min (or even 10 min) are used for the evaluation of [^18^F]fluorodopa conversion to ^18^F-fluorodopamine by aromatic amino acid decarboxylase and uptake and trapping of ^18^F-fluorodopamine into synaptic vesicles.

#### Image reconstruction

1. Iterative reconstructions are currently the standard. When Ordered-Subsets Expectation Maximization (OSEM) type methods are used, the number of iterations depends on the equipment characteristics, the injected activity, and the acquisition duration as well as on the corrections included in the reconstruction process.
2. Matrix sizes and zoom factors during reconstruction should consider the small size of the caudate and putamen. When possible, matrix sizes and zoom factors during reconstruction should be large enough in order that reconstructed voxel sizes are within 2.0–3.0 mm in any direction.
3. Regular corrections are necessary before or during image reconstruction such as attenuation, scatter, random, dead time and decay corrections, and sensitivity normalization. To date, the benefit of time-of-flight correction has been modest, but it can be applied [117, 118].
4. Resolution modeling during reconstruction, called point-spread function (PSF) reconstruction, deserves specific consideration. Resolution modeling has been developed to compensate for cross-contamination between adjacent functional regions with distinct activities, referred to as PVE [119]. In theory, given the size of the striatal structures, it would be beneficial to reconstruct the exact activity distribution using this correction. However, there is a limit to the recovery achievable by PSF correction, even at high iterations due to the loss of high frequency information during data acquisition [119]. The application of PSF correction modifies the noise structure and it produces a “lumpiness” aspect [120] (referred to as the Gibbs phenomenon). Therefore, PSF reconstruction artifacts can lead to misinterpretations when used to analyze small subcentimeter structures and is currently not recommended for striatal imaging
5. Spatial filters applied during or after reconstruction should be selected as not to impair the spatial resolution needed for striatal imaging. However, the choice is highly dependent on the acquisition data (noise) and the entire reconstruction process. Ideally, the final reconstructed spatial resolution should aim at a FWHM < 6 mm.
6. Head-movement correction can be performed if the acquisition is performed in list mode or multiframe setting (dynamic scans). Data acquired during patient movement can be discarded before reconstruction or corrected post reconstruction with dynamic or list mode acquisitions.

#### Interpretation/semiquantification

The uptake of [^18^F]fluorodopa over the first 90 min into the striatum primarily reflects influx and decarboxylation of the tracer to [^18^F]fluorodopamine [121].

##### Visual analysis

General concepts already discussed for visual analysis and interpretation of [^123^]FP-CIT SPECT also apply to [^18^F]fluorodopa PET images. See below further details and corresponding references specifically related to [^18^F]fluorodopa.

1. Images should be read on the computer screen since it is of paramount importance to adjust the color scale appropriately. Indeed, the first step of visual reading is the setting of the maximum color scale value. The reader should look for the voxel of maximal value within the striatum and select this value as the maximum of the chosen color scale. The position of this maximal uptake is important for the interpretation (see below).
2. Care should be taken in the 3D alignment of images to insure the proper appreciation of real uptake asymmetry. It is recommended to visualize all the transaxial slices containing striatal uptake as well as those including midbrain in order to be able to detect extra-striatal uptake.
3. Normal [^18^F]fluorodopa striatal uptake is much less dependent on age than DAT tracers.
4. In normal subjects, the striatal uptake is homogeneous within the striatum and symmetrical. It is important to know that the local maximum of striatal uptake is normally located in the putamen. Extra-striatal uptake of [^18^F]fluorodopa due to monoaminergic innervation is low and normally undetectable.
5. The presence of an asymmetric pattern of reduced putamen and preserved caudate uptake is visually characteristic of PD, the low uptake level in the most affected putamen accurately separates IPD cases from healthy controls in published series [122]. Putamen uptake reduction starts from its posterior part creating a posterior-anterior gradient. It is very useful to identify the local maximum of striatal uptake, which is located in the caudate in that case.
6. In case of nigrostriatal degeneration, the midbrain extra-striatal uptake becomes more visible due to global reduction of striatal [^18^F]fluorodopa uptake. Catecholaminergic (locus ceruleus) and dopaminergic (substantia nigra) nuclei may demonstrate a “Mickey Mouse” shape in the midbrain [123].
7. In most of the cases, atypical parkinsonian syndromes cannot be separated from IPD. However, it is noteworthy that, in CBD and in PSP, the caudate sparing, which is characteristic of IPD, is often lost. In cases of vascular origin, localized defects corresponding to micro-infarctions can be detectable. Direct registration side-by-side reading with MRI scan help interpretation by showing the spatial correspondence of MR striatal lacunae and localized [^18^F]fluorodopa defects.
8. In essential tremor, psychogenic parkinsonism, and drug-induced parkinsonism, [^18^F]fluorodopa uptake is normal.

##### Semiquantification

1. A simple approach for quantitating tracer uptake is to compare the striatal signal with the signal in the occipital (SOR) or cerebellar cortex where little decarboxylation occurs [122]. This approach has been shown to sensitively detect the asymmetric putamen [^18^F]fluorodopa reductions present in IPD, and the SOR reductions correlate with ratings of disability.
2. When a dynamic acquisition is performed, it is possible to compute [^18^F]fluorodopa influx constants (K_i_) from the brain time-activity curves (TACs). In fact, parametric images of specific [^18^F]fluorodopa influx constants (K_i_ maps) can be created at a voxel level for the whole brain using linear graphical analysis of TACs with an occipital cortex nonspecific reference input function [124]. The K_i_ is the product of the free [^18^F]fluorodopa brain volume of distribution (Vd) at equilibrium, and the decarboxylation rate constant k_3_ [125, 126]. Basically, K1 is thought to reflect [^18^F]fluorodopa transport from arterial plasma over the blood brain barrier into the dopaminergic neurons and the decarboxylation of [^18^F]fluorodopa to [^18^F]-dopamine [125, 126]. K_i_ is the product of the free [^18^F]fluorodopa brain volume of distribution (Vd) at equilibrium and the decarboxylation rate constant k_3_ [126, 127]. Ideally, this graphical approach requires a metabolite corrected plasma input function. Nevertheless, a reference region like occipital cortex (or cerebellum) where only minimal amounts of specific [^18^F]fluorodopa metabolites are assumed to exist (due negligible decarboxylation) can be used as a measure of plasma free [^18^F]fluorodopa signal avoiding blood sampling [127]. The nonspecific activity concentration is further assumed to be identical in the striatum and in the occipital cortex. The reference tissue derived influx constant K_i_ from brain uptake over 30–90 min then becomes the product of the [^18^F]fluorodopa decarboxylation rate constant k3 and the ratio of free brain [^18^F]fluorodopa / ([^18^F]fluorodopa +[^18^F]fluoromethyldopa) Vd.
3. In practice, K_i_ derived using the reference region approach and simple ratios of striatal/occipital fluorine-18 uptake at 90 min are highly correlated, and both approaches differentiate early PD from healthy controls. Either approach can be used for quantitation, although only few studies have to-date simplified K_i_ method with full kinetic analysis.
4. An advantage of the K_i_ over the ratio approach is that it provides a more direct measure of the [^18^F]fluorodopa decarboxylation rate constant.
5. Part of [^18^F]fluorodopamine is subsequently metabolized to [^18^F]FHVA (6-[^18^F]fluorohomovanilic acid) and [^18^F]FDOPAC (3,4-hidroxy-6-[^18^F]fluorophenylacetic acid). Accordingly, a dynamic PET scan at 3–4 h after [18F]fluorodopa administration reflects signal washout after 90 min due to combined MAOB and central COMT activity. PET can therefore be used to measure dopamine turnover in the striatum as a ratio of signal ^18^F loss (k_loss_) to influx K_i_ [127]. This approach, while potentially more sensitive than isolated K_i_ or SOR ratio measurements, requires scans at 90 min and again 3–4 h for the subject so is currently a research rather than a clinical tool [128].

#### Equipment specifications

7. State-of-the-art 3D PET/CT or PET/MRI systems should preferably be used. The equipment should allow for the acquisition of data needed for attenuation and scatter correction. Low-dose CT is the preferred option, although PET/MRI can use MRI sequences dedicated to attenuation and scatter correction of PET emission data. Transmission sources are also capable of producing adequate attenuation maps on dedicated brain PET only systems.
8. The equipment should have an axial field of view > 15 cm to assure sufficient coverage of the entire brain, including the cerebellum and brain stem.
9. The PET camera should be able to acquire both static and dynamic and frame or list mode PET emission data in 3D mode.

#### Quality control and interinstitutional PET system performance harmonization

The present guidelines are focused on the use of dopaminergic imaging in parkinsonian syndromes. However, there are both technical and imaging physics related uncertainties that apply to any PET examination regardless of radiotracer or specific application. These aspects have been detailed in previous guidelines [129]. These recommendations should be considered for the use of brain PET examinations in multicenter studies and/or when data are compared with a reference database or disease patterns. In order to guarantee sufficient image quality, quantitative performance, and image harmonization, the correct performance of the PET system must be regularly checked by several QC experiments that have also already previously listed in EANM and joint EANM/SNMMI guidelines. Cross-calibration of the PET(/CT) system against the locally used dose calibrator to prepare and measure patient-specific radiotracer activities is needed. Cross-calibrations should be performed following EARL recommendations and criteria (http://earl.eanm.org).

## Documentation and reporting for presynaptic dopaminergic imaging with either SPECT using iodine-123-labeled dopamine transporter ligands or with PET using [^18^F]fluorodopa

### Reporting

Patient’s name and other identifier (date of birth, name of the referring physician(s), type and date of examination) and patient’s history including the reason for requesting the study are mandatory parts of the report.

#### Body of the report

##### Procedures and materials

1. A brief description of the imaging procedure needs to be included. Details to be provided: radiopharmaceutical and administered activity, scan acquisition delay, and quality of the scan (the reason should be given if the quality is suboptimal due to motion artifacts).
2. If sedation is performed, a brief description with details on type and time of medication with respect to radiotracer injection should be provided (at least in theory, halogen-based anesthetics and benzodiazepines can increase endogenous dopamine levels).

##### Findings

1. Visual impression of striatal binding compared with background activity should be provided with details on both the caudate nuclei and the putamina. Note any significant asymmetries; mild asymmetry may occur in healthy individuals. If abnormalities are present, location and intensity of this reduction should be provided. Signal to noise ratio has to be commented. The visualization of extra-striatal uptake should be reported. Criteria used for interpretation should be described (visual versus semiquantitative assessment or comparison with normal database).
2. If semiquantitative analysis for DAT SPECT is performed, report the values. If a normal database for patients’ value comparison, report reference range and/or z-score differences with respect to the database in case of abnormal findings (see discussion of utility and limits of database of healthy subjects in previous sections).
3. Comment findings on the light of relevant anatomic changes displayed on morphological imaging (CT or MRI scans).
4. Limitations: list factors that could have limited accuracy of the examination in terms of sensitivity and specificity (i.e., movement or concomitant medication). Report potential interference due to drugs administered to the patients.
5. Clinical issues: the report should address or answer any pertinent clinical issues raised in the request for the imaging examination.

##### Comparative data

Comparisons with previous examinations and reports, if available, should be part of the report indicated. However, in view of the high sensitivity and early impairment characterizing presynaptic dopaminergic imaging, a follow-up scan is rarely needed or indicated.

#### Interpretation and conclusions

##### Conclusions

The report should include conclusions stating if a presynaptic dopaminergic deficit is present or absent. Abnormal findings indicate a presynaptic striatal dopaminergic deficit. This finding might be due to a variety of diagnoses, including IPD, PSP, MSA, CBD, and DLB. When caudate sparing characteristic of IPD is lost, diagnoses of CBD or PSP can be suggested, especially with [^18^F]fluorodopa, which allows higher spatial resolution. Normal findings could support the diagnosis of essential tremor, drug-induced parkinsonism, psychogenic parkinsonism, AD, or a state of health. To aid the referring clinician, descriptors such as mild, moderate, or severe can be used to characterize any deficits. A more comprehensive diagnostic comment taking advantage of the available results of other previously performed molecular (i.e., ([^18^F]FDG PET or [^123^I]mIBG) or structural imaging studies (i.e., MRI with a punched-out appearance of the striatum) could be included in selected cases. Similarly, when appropriate, follow-up or additional studies ([^18^F]FDG PET, perfusion SPECT, cardiac [^123^I]mIBG scintigraphy, or D2 imaging) can be recommended to clarify or confirm the suspected diagnosis.

##### Sources of error and artifacts

1. Artifacts (patient movement, camera-related, induced by inappropriate processing; tracer extravasations).
2. Interference from medications (although interactions should affect tracer uptake in the whole striatum and should not influence the presence of an asymmetrical pattern of reduced uptake).

## New tracers for dopaminergic imaging in Parkinsonian syndromes

### PET imaging of DAT using ^11^C-PE2I and ^18^F-FE-PE2I

Among several DAT radioligands available for PET imaging, [^11^C]-PE2I (N-(3-iodopro-2*E*-enyl)-2β-carbo[^11^C]methoxy-3β-(4′-methylphenyl)nortropane) was developed in early 2000 in order to image and quantify the DAT not only in the striatum but also in extra-striatal regions, such as the substantia nigra [130–132]. [^11^C]PE2I is highly selective for the DAT and displays high target-to-background ratio and good test-retest reliability [133]. It has been already used in the context of clinical trials and PET studies in patients with PD and in animal models of parkinsonism [134–138].

Despite the suitable properties as a PET radioligand for the DAT, absolute quantification requires long duration of imaging for at least 90 min due to slow wash-out from the brain [139]. In addition, a radioactive metabolite of [^11^C]PE2I has been found to cross the blood-brain barrier and accumulate in the striatum in rodents. Therefore, further efforts have been made in order to develop an analog of [^11^C]PE2I with improved imaging properties. [^18^F]FE-PE2I (N-(3-iodoprop-2E-enyl)-2β-carbo[^18^F]fluoroethoxy-3β-(4-methylphenyl)-nortropane) is a fluoroethyl analog of [^11^C]PE2I developed at Karolinska Institute and highly selective for the DAT with improved pharmacokinetic properties compared with [^11^C]PE2I: (i) faster washout from the brain and (ii) lower production of a radiometabolite (< 2% of plasma radioactivity) that crosses the blood-brain barrier [140–143].

The quantification can be done using the cerebellum as reference region [143]. A simplified method for the quantification of the SBR can also be used by acquiring a static image around the peak specific binding between 16.5 and 42 min after injection [144].

The affinity of [^18^F]FE-PE2I for the DAT (K_i_ = 12 nM) and the high target-to-background ratio permit to quantify the DAT in the striatum and in the substantia nigra [142–145]. Compared with other tropane analogs including ß-CIT and FP-CIT, FE-PE2I is by far more selective for the DAT than for other monoamine transporters [130].

Initial studies have shown that in early PD, DAT is decreased by approximately 70% in the putamen and 30% in the substantia nigra, indicating a preferential loss of the DAT in the axonal terminals [145].

The dosimetry of [^18^F]FE-PE2I has been estimated in nonhuman primates and human subjects [146, 147]. The estimated effective dose (ED) is 0.023 mSv/MBq, which is similar to that of other ^18^F-labeled radioligands for brain imaging [148].

Further studies with [^18^F]FE-PE2I are ongoing to examine the DAT loss across different stages of IPD, the test-retest reliability, clinical most appropriate imaging interval, and the longitudinal assessment in IPD patients. Head-to-head comparative studies with [^123^I]FP-CIT are also ongoing to examine the relative performance of [^18^F]FE-PE2I in differentiating IPD from controls. The first published comparative study reported that binding potential (*BP*_ND_) of [^18^F]FE-PE2I was highly correlated with [^123^I]FP-CIT data, with intraclass correlation coefficients > 0.9 [148]. Similar effect sizes were reported for both radioligands, indicating that [^18^F]FE-PE2I has similar capability to differentiate patients with parkinsonism from controls as [^123^I]FP-CIT [148].

Further studies (and probably long term follow-up) will be needed to assess if patients with uncertain borderline [^18^F]FE-PE2I PET that cannot be explained by structural change/lesions can be better separated based on high or low uptake in the substantia nigra.

The primary clinical benefits compared with DAT SPECT include the improved resolution of PET, an improved clinical workflow, patient and caregiver convenience as a thyroid-blocking agent is not required, and shorter injection-to-scan and acquisition times.

Radiopharmaceutical’s characteristics

1. Administered activity to adults: 100–250 MBq (typically 200 MBq)
2. PET data acquisition:
  Suggested dynamic acquisition for estimation of binding potential (*BP*_ND_): duration, 60 min
  Suggested static acquisitions: for best simplified estimation of specific binding ratios: 25 min from 17 to 42 min.; for routine clinical use: 10 min from 30 to 40 min
3. Image processing:

Measured or CT attenuation correction

OSEM reconstruction: resolution/PSF modeling not needed for routine clinical use. Final image resolution: 5–8 mm

4. Quantification

Binding potential (*BP*_ND_) estimated with simplified reference tissue method or Logan graphical analysis using cerebellum as reference region [143].

5. Semiquantification

SBR calculated with cerebellar gray matter as reference. Target metrics: SBR Caudate, SBR Putamen, SBR Striatum; Putamen/Caudate ratio; Hemisphere asymmetry index = (R − L)/(R + L)

### PET imaging of VMAT2

After synthesis, monoaminergic neurotransmitters are concentrated in synaptic vesicles for exocytotic release. The vesicular monoamine transporter type-2 (VMAT2) is expressed by all monoaminergic neurons and serves to transport transmitter from cytoplasm into vesicles [149]. In the CNS, VMAT2 is expressed exclusively by monoaminergic (dopaminergic, serotonergic, norepinephrinergic, or histaminergic) neurons [150], albeit it is also expressed in pancreatic beta cells and several monoaminergic neurons of the human enteric nervous system [151]. Over 95% of striatal VMAT2 binding sites are associated with dopaminergic terminals [152]. In vivo imaging and quantification of VMAT2 has been reported for a series of radioligands based on the vesicle-depleting drug tetrabenazine. The most widely used ligand for research purposes is (+)-[^11^C]dihydrotetrabenazine ([^11^C]DTBZ), which binds specifically and reversibly to VMAT2 and is amenable to quantification of striatal, diencephalic, and brainstem neurons and terminals with PET [153, 154]. As only the (+)-enantiomer binds specifically to VMAT2, use of the racemic tracer reduces specificity with ensuing lower image contrast. Despite the successful application of (+)-[^11^C]DTBZ for human studies, clinical utilization will likely require radioligands with longer half-lives. Fortunately, development of fluorine-18-labeled VMAT2 radioligands such as (+)-[^18^F]FP-DTBZ ((+)-α-9-O-(3-[^18^F]fluoropropyl)DTBZ) provides a ^18^F-labeled VMAT2 ligand that will be more suitable for routine use in clinical practice [155]. When compared with (+)-[^11^C]DTBZ, it is not only more favorable due to the longer half-life fluorine-18 labelling but also has higher affinity for the VMAT2 receptor (K_i_ = 0.3 nM vs. 0.1 nM).

Radiopharmaceutical’s characteristics

1. Administered activity to adults: 300–450 MBq

2. Radiation dosimetry

Radioactivity uptake in the brain is highest at 7.5 ± 0.6% injected dose at 10 min after injection. High absorbed doses were found in pancreas, liver, and upper large intestine wall. The highest-dosed organ, which received 153 ± 24 μGy/MBq, was the pancreas. The effective dose for (+)-[^18^F]FP-DTBZ was 28 ± 3 μSv/MBq. These values are comparable with those reported for other fluorine-18 labeled radiopharmaceuticals [155].

3. PET data acquisition

A single 10-min PET scan can be acquired 90 min postinjection in 3D mode. Scanning time of 90–100 min for (+)-[^18^F]FP-DTBZ is considered as the optimal time window for summed uptake measurements in terms of correlation of standardized uptake value ratio (SUVRs of striatum to occipital cortex) to distribution volume ratios (obtained with dynamic acquisition) as well as in terms of differential power, stability, and clinical feasibility across and between patients with IPD and control subjects [156].

4. Image processing
  Measured or CT attenuation correction
  PET images can be reconstructed using 3D OSEM algorithm
5. Visual assessment

(+)-[^18^F]FP-DTBZ binding in normal control subjects shows symmetric and highest uptake of (+)-[^18^F]FP-DTBZ in striatum, followed by nucleus accumbens, hypothalamus, substantia nigra, and raphe nuclei [157].

6. Quantification analysis
  Striatal VMAT2 density of anterior putamen is higher than posterior putamen, which is higher than that of the caudate nucleus. Lowest uptake is seen in the cortex with essentially no specific binding in the occipital cortex [157].

## Procedure/specifications of the examination for postsynaptic dopaminergic imaging

Historically, the most widely applied radiotracers for imaging D2-like receptors with SPECT has been [^123^I]IBZM [158, 159]. For PET, [^11^C]raclopride, [^18^F]fallypride and [^18^F]Desmethoxyfallypride (DMFP) have been used. These dopamine receptor antagonist derivatives are not selective radiopharmaceuticals for the D2 receptor since they also bind to the D3 receptor [159]. However, the vast majority of D2-like receptors in the striatum are D2 receptors. EANM GLs aiming to describe standard procedures for brain neurotransmission using dopamine D2 receptor ligands have been published in 2009 [9]. These guidelines dealt with indications, assessment, processing, interpretation, and reporting of D2 imaging. The procedural recommendations defined in these GLs are still presently valid with the exception of details on equipment specification, which should be updated in agreement of what already described in previous paragraphs of the present GLs. It should be noted that the main added clinical value of D2 imaging in patients with parkinsonian syndromes was the proposed capability of this imaging to support the differential diagnosis within neurodegenerative parkinsonisms by differentiating between diseases with (i.e., atypical parkinsonism such as MSA, CBD, and PSP) and without (i.e., IPD) degeneration at postsynaptic level [10]. However, D2 SPECT imaging is at the moment not clinically available in several countries, and it has been suggested that FDG PET outperforms D2 SPECT Imaging for the differential diagnosis between PD/DLB and the other atypical parkinsonisms [27]. Similarly, [^123^I]mIBG myocardial scintigraphy can be used to differentiate between IPD and MSA, PSP and CBD [160]. Accordingly, updated data on the use of these tracers are beyond the aims of the present clinical use-oriented standard procedures, and the procedural published by the EANM NeuroImaging Committee in 2009 should be still considered as the main reference for D2 dopaminergic imaging in parkinsonian syndromes [9].

## Radiation safety

In this section, we present a set of values and advice that are compliant with the legal requirements in the European Basic Safety Standards Directive and with IAEA Safety Guides and the SNMMI Guideline for General Imaging [161, 162]. However, the way the EU directive is being implemented in the national member states in Europe varies considerably. In addition, regional and local rules, e.g., from local ethical committees, may also apply in a specific setting and supersede this guideline. The general system of radiation protection is based on the three concepts of (1) justification, (2) optimization, and (3) dose limits.

Justification for an examination at the individual level is implicit when following the indications in this guideline. The set of acquisition parameters given in this guide serves to optimize the activity used (and the dose provided) for current standard equipment. Further local optimization may apply when special, more sensitive equipment is available. Dose limits do not apply for medical exposure of patients; protection is managed through the justification and optimization only. For biomedical research performed on patients or volunteers, however, ICRP (ICRP62 and ICRP103), WHO, and EC (EC RP99) have issued guidance balancing the value for society of the expected outcome of the research against (effective) dose. This is the source of information that ethics committees (institutional review boards) will typically consider.

In addition—since medical use of radiation is a “planned exposure”—the concept of dose constraints applies to “comforters and cares,” to relatives, to “other” hospital staff not working directly with ionizing radiation, and to members of the public. The relevant radiation exposure for these groups is highly dependent on local factors and procedures. It therefore falls outside the scope of this guideline and must be handled by the associated medical physics expert.

For the tracers described in this guideline, effective dose and absorbed dose (per MBq) to the highest exposed organs has been compiled from the available literature and is given in Table 4 [40, 147, 155, 163–169]. In most cases, following this guideline will lead to effective doses below 5 mSv per examination.

**Table 4:** Radiation dosimetry (in adults)

Radiotracer	Organs receiving the highest absorbed dose	mGy/MBq	Effective dose (mSv/MBq)	Reference
[^123^I]β-CIT	Lung	0.10	0.031–0.04	[40, 163, 164]
	Liver	0.09		
	Basal Ganglia^*^	0.27^**^		
[^123^I]FP-CIT	Liver	0.085	0.025	[165]
	Colon	0.059		
	Gall bladder wall	0.044		
	Lung	0.042		
[^99m^Tc]Trodat-1	Liver	0.047	0.012	[166]
	Kidney	0.035		
[^18^F]-FDOPA	Urinary bladder wall	0.30	0.025	[165]
	Kidney	0.031		
[^123^I]-PE2I	Urinary bladder wall	0.07	0.022	[167]
[^11^C]-PE2I	Urinary bladder wall	0.018	0.0064	[168]
	Kidney	0.016		
	Stomach wall	0.014		
[^18^F]FE-PE2I	Urinary bladder wall	0.119	0.023	[147]
	Liver	0.046		
[^11^C]DTBZ	Stomach wall	0.016	0.0066	[169]
	Kidney	0.013		
[^18^F]FP-DTBZ ([^18^F]AV-133)	Pancreas	0.153	0.028	[155]
	Liver	0.072		
	Upper large intestine	0.055		

When a review performed by the International Commission for Radiological Protection (ICRP) was available, this reference was included (ref 162). In other cases, it should be noted that reference may be related to one single article. It should be noted that the human dosimetry for the tracers presented in the table in most cases is based on studies including a very limited number of patients/volunteers. The (high) precision of the numbers shown does not necessarily reflect the actual accuracy of the information. Uncertainties reported in the literature are usually the variation observed in the material but does not include systematic errors in the methods applied

*The basal ganglia is normally not considered “an organ” in the ICRP context. The value is given here because it is significantly higher than for other tissues, and posted in the reference cited

**Values in the reference is given separately for the two genders. Here the average is presented

If a CT scan is utilized for attenuation correction only, the lowest possible exposure settings should be used, resulting in a contribution to effective dose of less than 0.1 mSv.

In radiation protection, there is a special concern for a fetus (pregnancy) and small children (breastfeeding) due to their higher radiosensitivity. General rules for obtaining information about pregnancy status must be established and followed as locally implemented. Neither pregnancy nor breastfeeding are absolute contraindications for imaging using ionizing radiation, but require careful considerations for the justification. The need to support the diagnosis of parkinsonian syndromes with SPECT or PET examinations rarely apply to pregnant or breastfeeding patients.

However, like for any other diagnostic procedure in a female patient known or suspected to be pregnant, a clinical decision is necessary in which the benefits are weighed against the possible (hypothetical) harm. If the procedure consists of a static scan, it may be possible to reduce the activity (hence the absorbed dose to organs and effective dose) and prolong the acquisition time to maintain image quality; a reduction in image quality is not recommended.

### Breastfeeding

Before administering radiopharmaceuticals to a mother who is breastfeeding, consideration should be given to the possibility of delaying the examination (administration of radionuclide) until the mother has ceased breastfeeding and to what is the most appropriate radiopharmaceutical choice, bearing in mind the possible secretion of activity in breast milk. Unfortunately, since these events are rare, the data available about this secretion is also sparse and often uncertain. Formally (in the terminology of radiation protection) the child is a “member of the public,” and therefore, the protection of the child is based on a dose constraint of 1 mSv (ICRP103, BSS, IAEA). This dose constraint is the combined result of an intake of radioactivity and a contact dose. Ideally, these two sources should be considered separately since contact dose might be eliminated if the mother expresses milk that can be given to the child by a third person. The recent IAEA Safety Guide, however, only gives recommendations that include both sources and are obviously different for tracers labeled with ^123^I, ^11^C, or ^18^F (crf IAEA Safety Guide, Appendix III) [162].
